# Special Issue: “Plant Virus Epidemiology”

**DOI:** 10.3390/plants10061188

**Published:** 2021-06-11

**Authors:** Dirk Janssen, Leticia Ruiz

**Affiliations:** IFAPA, Centro La Mojonera, 04745 La Mojonera, Spain; mleticia.ruiz@juntadeandalucia.es

We are pleased to present this Special Issue on the topic of “Plant virus Epidemiology”. This issue includes a series of articles on important aspects related to plant viruses. Plant diseases in general are in fact both fascinating and abominable. Although the consumer usually perceives the effect of these diseases only in an anecdotal way, like when the fruit and vegetables in the home spoil, the truth is that they have an extraordinary impact on the life and health of humans and livestock. This impact has profoundly marked the history of humanity, since it is only a little more than a century and a half ago that we have begun to unravel the biological bases of diseases, understand their causes, and mitigate their effects. Among the pathogens that cause them, viruses are admirable weapons of mass destruction. On the one hand, they are a paradigm of biological simplicity that places them on the frontier of life. However, they are also the second group of plant pathogens and they currently cover some 1516 species across 26 families (10th Report of the International Committee on Taxonomy of Viruses (ICTV2018b.v1)). Plant viruses are also economically important, and, in general, this increases proportionally with the intensification of agriculture [1,2]. This Special Issue contains a comprehensive overview on virus disease pandemics or major epidemics that are known to have, or considered likely to have, originated within crop domestication centres in different parts of the world, that became distributed from there to other continents, and now have global distributions. The examples described infect cereals (maize, rice, wheat), root and tuber crops (potato, sweet potato), plantation and orchard crops (banana, citrus, stone fruit), grain legumes (faba bean), and annual horticultural crops (tomato, cucurbits). Both historical and recent information is provided about eight examples of virus diseases that threaten staple food crops and, therefore, are of critical importance regarding global food security [3]. Two of the viruses presented in this review as the cause of major epidemics, are tomato brown rugose fruit virus (ToBRFV) and pepino mosaic virus (PepMV) which are also subject of another research paper in this Special Issue [4].

The possibilities of acting directly on viruses are less than for other pathogens that cause losses, and their control is fundamentally based on two strategies: (i) control of inoculum sources (e.g., sanitation of host propagation material, control of vector populations, elimination of alternative hosts), and (ii) the use of resistant cultivars, obtained by conventional genetic improvement or by genetic engineering methods. Therefore, it is essential to have a thorough knowledge of the epidemiology of diseases caused by viruses, which allows the best use of the control tools available. The epidemic is defined as the variation, in time or space, of the amount of diseases in a host population. In general, the definition of epidemic substitutes variation for increase, but variation reflects a greater number of situations that can occur throughout the development of an epidemic [5]. In the middle of the 20th century, the transition from qualitative to quantitative epidemiology took place, with Vanderplank [6] being one of the main responsible in this field. Since then, different mathematical models have been applied to epidemics caused by plant pathogens that allow them to be compared, study the effect of different control methods or environmental conditions on their development, and help predict them [7]. The use of models with a different mathematical approach has allowed not only to quantify the temporal and spatial evolution of epidemics caused by viruses, but to compare, analyse, and identify the factors that most influence their progress. It must also be taken into account that with the exception of potexviruses and tobamoviruses, the viruses that cause major epidemics in crops are transmitted by some type of vector (arthropods, fungi, nematodes, etc.). For this reason, epidemiological analyses must not only consider the dynamics of the virus population, but also that of the vector population, the virus-vector interactions and how the different environmental conditions of the environment (including the host) affect both populations and, therefore, the development of the epidemic. This could represent the conventional view of plant virus disease epidemiology. However, the recent technological developments in plant virus epidemiology research offer new opportunities for further synthesis by a greater consolidation and extension of ecological and evolutionary insights into epidemiological analysis of the causes and consequences of plant virus disease epidemics; in particular, by recognising the shorter-term (ecological) and longer-term (evolutionary) consequences of disease epidemics. This new synthesis that joins conventional epidemiology with virus origin, evolution, and host range, must include ecological factors and presents new challenges for plant virus research [8]. It is argued that in the synthesis of epidemiology, evolution, and ecology, there should be a greater recognition that domesticated crop plants, even in extensive monocultures and other restricted cropping systems with less biodiversity also have an ecology, not just the wild or relatively unmanaged plant communities which are often used as examples for an ecological approach. A fewer number of studies have attempted to extend the analysis globally. An example is given for the Solanaceae family, which has species in all the above settings, is present globally, and is affected by a wide range of viruses [9]. The authors of this paper in this Special Issue concluded that new disease management practices and diagnostic methods would be needed to cope with the global changes affecting the agricultural–environmental interface, by targeting the entire Solanaceae community.

Breeding for disease resistance plays a major role in developing strategies for disease management. This is especially the case for plant virus disease. Cucumber green mottle mosaic virus (CGMMV) is a severe threat to melon production worldwide. At present, there are no cultivars available on the market which show an effective resistance or tolerance to CGMMV infection. Different strains of this virus exist in different parts of the world, and recently strains of putative Asian origin have entered Spain, coinfecting and subsequently displacing the European strain. It was found that *C. melo* accessions behave different following the inoculation with either one or the other virus strain. These results are important for melon breeders, but help to explain the recent successful spread of the Asian CGGMV strain in Spain and many other countries in the world [10]. As with other pathogen groups, less attention has been given to how host resistance should be used in the field. By comparison little has been done on the transmission and spread of plant viruses in resistant varieties under field conditions. From an epidemiological perspective, this is a major gap that is holding back progress in disease management and will be emphasised in a paper from this Special Issue. The epidemics of sweet potato virus disease (SPVD, caused by sweet potato feathery mottle virus and sweet potato chlorotic stunt virus) were monitored over time and with leaf profile for 10 sweet potato varieties covering a range of resistance characteristics. The data provided detailed information on the development of SPVD and the molecular responses that were occurring in the field based on transcriptomics. It was found that resistance was characterised both by disease incidence on a per plant basis, but also by the number of symptomatic leaves per infected plant. Such an approach can provided invaluable information to guide molecular breeding approaches, and further how the developed varieties can best be deployed in the field [11].

The epidemiology of plant viruses becomes more complicated in systems where several virus species are involved. Watermelon mosaic virus (WMV) and Moroccan watermelon mosaic virus (MWMV) infect cucurbitaceous crop species like melon, watermelon, and zucchini. However, a monitoring of crops revealed the different presence of WMV and MWME which could be explained on host plant species, but also on the different ranges of plant host species, and of aphid vector species between the two virus species. In addition, experimental infections suggest that mixed infections with both virus species in the same host also determines the viral loads, and may explain impaired distribution of one of the viruses [12].

Mixed infections of different virus species is also studied in another paper in this Special Issue, and this involves two important emerging viruses in tomato [4]. Since the first description of ToBRFV the control of the disease caused by this species represent one of the mayor challenges in horticulture. However, another emerging virus, PepMV, widely distributed in tomato crops in many countries in the world, could play an important part in the symptoms observed in infected crops. No commercial resistant tomato varieties are currently available. Importantly, the paper reports on biological assays testing the contribution of traded infected tomatoes to the establishment of tomato plant disease. The results, showed that the ToBRFV and PepMV co-infected fruits were an effective inoculum source for disease spread only when fruits were damaged. In contrast, intact fruits did not spread the viral disease. These results added a new factor to disease epidemiology of these viruses [4].

We hope that the articles in this Special Issue on “*Plant Virus Epidemiology*” highlight elaborate efforts in modern plant virus epidemiology and that they support a broader view of the complex nature of how plant diseases caused by viruses occur and offer clues to its control. In closing, we thank all of the authors for their enlightening contributions to this Special Issue.
